# The mosaic structure of the mammalian cognitive map

**DOI:** 10.3758/s13420-023-00618-9

**Published:** 2024-01-17

**Authors:** Kate J. Jeffery

**Affiliations:** School of Psychology and Neuroscience, College of Medical, Veterinary and Life Sciences, University of Glasgow, Glasgow, UK

**Keywords:** Spatial behaviour, Spatial cognition, Cognitive map, Hippocampus, Sense of direction, Place cells, Grid cells, Head direction cells

## Abstract

The cognitive map, proposed by Tolman in the 1940s, is a hypothetical internal representation of space constructed by the brain to enable an animal to undertake flexible spatial behaviors such as navigation. The subsequent discovery of place cells in the hippocampus of rats suggested that such a map-like representation does exist, and also provided a tool with which to explore its properties. Single-neuron studies in rodents conducted in small singular spaces have suggested that the map is founded on a metric framework, preserving distances and directions in an abstract representational format. An open question is whether this metric structure pertains over extended, often complexly structured real-world space. The data reviewed here suggest that this is not the case. The emerging picture is that instead of being a single, unified construct, the map is a mosaic of fragments that are heterogeneous, variably metric, multiply scaled, and sometimes laid on top of each other. Important organizing factors within and between fragments include boundaries, context, compass direction, and gravity. The map functions not to provide a comprehensive and precise rendering of the environment but rather to support adaptive behavior, tailored to the species and situation.

## The cognitive map

The concept of the cognitive map was proposed in the 1940s by Tolman, who formulated it on the basis of his experimental observations of maze-learning in rats. He noted that animals often display behaviors suggesting knowledge of the environment layout, and wrote: “We believe that in the course of learning something like a field map of the environment gets established in the rat's brain” (Tolman, 1948). With hindsight, the “field map” analogy was unfortunate because it implies a two-dimensional sheet-like representation, which is inconsistent with what we know of brain anatomy and neuronal function, and seemed implausible to many researchers. We now understand “map” in the more abstract sense of a pattern of neural activity in which there is a correspondence, or isomorphism (Gallistel, 1989, 1990), between neural activity and the real world, such that operations in the neural system can be used to make inferences about the world (e.g., the shortest path from A to B). Later, we will explore in more detail what is meant by “map” and whether (if cognitive maps exist) there is *a* map or many.

**Fig. 1:**
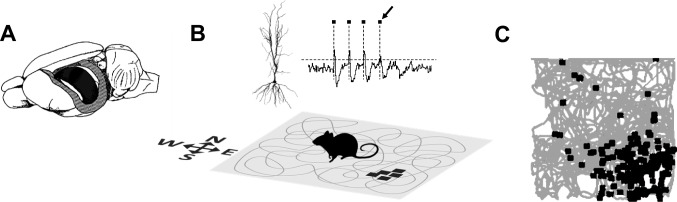
Recording a place cell. (**A**) Schematic of the rat brain, with the outer neocortex on the left side cut away to reveal the hippocampus underneath (black cashew-shaped structure). (**B**) The recording setup for place cells. The animal explores a space, such as this square arena, while neurons are recorded from the dorsal region of the hippocampus. The path of the animal is shown as a grey line. The insets show a typical pyramidal neuron with dendritic arbors reaching both upwards and downwards, and a typical segment of recording as would be seen on an oscilloscope. The action potentials fired by the neuron are visible as “spikes” against the baseline, marked here with small squares (the arrow points to a single spike). Each black square on the arena indicates the location of the rat at the moment when that spike was emitted. (**C**) How the data from a recording trial are depicted. The grey line represents, as in (**B**), the path of the animal over about 10 min of exploration, while the black squares indicate the locations of the spikes that were recorded from a single neuron. Note that the spikes tended to be emitted in one region of the environment. This region is called the place field of the cell

When O’Keefe and Dostrovsky (1971) first reported observations of spatially localized neuronal activity in the rat hippocampus, they claimed to have found Tolman’s cognitive map (O’Keefe & Nadel, 1978). These neurons, which O’Keefe named place cells, are active in their preferred locations, called place fields or firing fields, in a way that is stable over repeated visits to that environment (Fig. 1A), and does not depend on exact sensory inputs. For example, firing fields persist regardless of the facing direction of the animal (and hence what it can see) and they tolerate removal of landmarks or changes in lighting. This property led O’Keefe and Nadel to suggest that the function of these neurons is less sensory, to do with perception, and more cognitive, to do with knowledge.

**Fig. 2:**
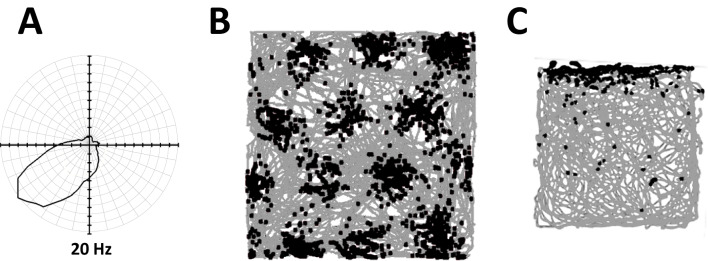
Head direction (HD) cell, grid cell, and border cell. (**A**) The activity of a single HD cell, plotted as firing rate as a function of head direction. This cell dramatically increases its firing rate when the animal faces in one particular direction but is almost silent in the other directions. (**B**) Grid cell, activity shown as in Fig. 1. Like the place cell, this cell fired in specific regions of the environment, but unlike the place cell these regions were multiple, uniformly circular and laid out in evenly spaced rows, forming a hexagonal close-packed tiling of the environment surface. Grid cells are mostly found in the medial entorhinal cortex, which is one of the major inputs to the place cell system. (**C**) Border cell, recorded from the medial entorhinal cortex

Following the discovery of place cells, a wealth of new findings subsequently confirmed that the brain indeed constructs an internal representation of space (see Moser et al., 2017, for a detailed historical review of these). The next major discovery was of directionally sensitive neurons, the head direction (HD) cells (Taube et al., 1990a; Fig. 2A). Each HD cell fires when the animal faces in a given direction. The directions may not be the same, globally speaking, in different environments, which indicates that the signal is not anchored by geomagnetic cues or any other distant cue: the determinant of direction is local, from the immediate environment, and seems to be predominantly visual. HD cells almost always fire coherently, which is to say that they maintain the same relative firing directions in every environment, although the absolute directions (relative to global North), may change. This coherence has been suggested to arise from so-called attractor dynamics (Skaggs et al., 1995; Zhang, 1996), in which interconnected cells coordinate their activity so that the collective output is a “consensus” of the decisions made by multiple elements of the network.

The third transformative discovery was a spatial cell type in a region upstream of hippocampus, medial entorhinal cortex (mEC), discovered in 2005 (Hafting et al., 2005), that were named grid cells. Grid cells produce place fields (Fig. 2B), but these differ in several important respects from hippocampal place fields. They are more circular, whereas hippocampal place fields are often oval or irregular. They are also multiple, whereas in a typical small laboratory enclosure a place cell only expresses one or two place fields. But most surprising of all is that the spacing between the firing fields is very uniform, with the consequence that the fields align themselves into neat rows, creating a remarkable hexagonal grid-like pattern (hence their name). Experiments manipulating sensory and motor cues have shown that this regular spacing and constant alignment results from the operation of path integration (Savelli & Knierim, 2019): the process by which spatial location is updated on a map by tracking movement in linear and angular domains. Indeed, the suggestion has been made that the grid cells *are* the brain’s path integrator (McNaughton et al., 2006), although there is reason to think this process is in fact more distributed.

The place, head direction, and grid cells provide a window into the internal machinery of the cognitive map. Below, we look at some of the major classes of information that the cells use to organize their firing patterns, before examining the question of how the activity of these neurons hints at the structure of the map.

## Organizing factors for the cognitive map

The mammalian cognitive map, as implemented by the spatial neurons described above, is a highly abstract notion that is useful for discussion but should not be taken to imply constraints on how the brain represents space based on our usual notion of what a map is. Rather, “map” is a useful shorthand for a highly complex interconnected set of processes that have, in common, a way for the organism to link its incoming sensory data to its location in the real world. Similarly, we do not commit at this point to the notion that there is necessarily one single map versus multiple maps, although later on will speculate that “the” map (meaning the sum total of the animal’s representation of its previously experienced spaces) has multiple related subcomponents.

When thinking about how “the” map is organized, a significant point to begin with is that the information that shapes the map is generally not in its raw sensory form: it has been combined across sensory modalities, with different combinations serving different organizational functions. We can think of these combined signals as having semantic content or “meaning” with respect to the map (sometimes more than one meaning). By “meaning” is meant some type of isomorphism with a spatially relevant variable, such as direction of movement or speed of movement, linking the internal neural activity with the real world. The organizational semantic categories we consider here are boundaries, context, compass direction, and gravity.

### Boundaries

Rodent place and grid cells use the environment boundaries to anchor their firing fields (Barry et al., 2007; O’Keefe & Burgess, 1996). This is shown by an experiment that investigated place cells while boundaries were progressively removed until the only remaining spatial cues were point-like poles (Barry et al., 2006). This resulted in the place fields breaking down even though, in principle, sufficient spatial information remained for triangulation of location. In another experiment, place fields were found to follow a local bounded environment (a small 1-m square recording box with walls low enough for the animal to see beyond) when this was moved within a larger room, provided the movement was small (a few cm) and not too repetitive (Hayman et al., 2003). However, with larger movements (a meter or more) and with repeated experience, place cells started to change their firing fields, generating an altered map of the space in a process previously called “remapping” (Muller & Kubie, 1987). Since in this experiment the only thing that changed was the spatial relationship between the box and the room, place cells must process this relationship. Interestingly, this processing was environment-specific – the same cell that “knew” about the two box locations when the box was black did not express this knowledge when the box was white. This suggests that information from the immediate boundaries can be decoupled from other types of information, even within the same cell (Jeffery & Anderson, 2003).

Exactly how boundaries are used by the cells has been explored by selectively manipulating subsets of them to see how the cells respond to the conflict this introduces. In one early experiment, Gothard et al. showed that moving an end wall along a linear track would cause place fields to shift along the track to maintain a relatively constant distance (Gothard et al., 1996), although the effect was stronger for fields closer to the wall, suggesting an additional influence of other cues: notably self-motion distance-tracking cues, which we discuss later. At around the same time, O’Keefe and Burgess (1996) recorded from rats exploring a box with moveable walls that was situated within a larger room. They found that when the box was stretched, place cells shifted and/or stretched their firing fields as if these were somehow linked to the moved walls. Notably, different cells followed different walls, so that the relative locations of their firing fields changed, thus providing perhaps the first indication that the place cell map is not rigid but can be deformed, and also that the cells act as individuals rather than a collective.

A subsequent experiment with grid cells found the same stretching phenomenon (Barry et al., 2007). This was slightly surprising, because if path integration is driving place cell distance-tracking, and this comes via the grid cells, one might expect the grid to remain rigid even when the walls are moved, since the scale is supposedly intrinsic to the cells. Thus, the distance-tracking input to the grid must be upstream of the grid cells, and compete with whatever process allows the grid to attach to the boundaries (discussed later). Notably, in both the place and grid cell experiments the amount by which fields followed the walls was only around 50% of the actual wall displacement, indicating some kind of compromise between the influence of the walls and that of the distance-tracking self-motion signal. 

The responsiveness of grid cells and place cells to environmental stretching suggests that the borders of the environment anchor the map: that is, they provide the references against which travel distance is measured. For this to work, the system needs to be able to link a given border with its direction relative to the environment center. O’Keefe, Burgess, and colleagues proposed that this is accomplished by means of putative boundary vector cells (Hartley et al., 2000), each of which is sensitive to a border located at a configural combination of distance and direction from the animal. Note that this is an “allocentric” (world-centered) signal: the animal itself can be facing in any direction, and so the border can be located in any *ego*centric (animal-centered) direction from the animal (ahead, behind, to the left, etc.), provided it has the requisite global direction. In rodents, cells with these proposed boundary vector properties have since been reported in two brain regions: subiculum, in which the firing is broad, and located at varying distances from the border (Lever et al., 2009), and mEC, in which the firing tends to be up against the environment walls (Solstad et al., 2008). These two cell classes tend to be referred to as boundary vector cells and border cells (Fig. 2C), respectively, although their properties have not been fully elucidated as yet. Border/boundary-vector cells may anchor the spatial map via co-activation with place or grid cells whose fields lie near the borders. In support of this, contact with boundaries is able to correct drift errors in grid cells (Hardcastle et al., 2015). Boundary vector coding has also been recently reported in fish (Cohen et al., 2023).

Because the sensory information concerning the allocentric direction of a border arrives in an egocentric reference – i.e., via the senses, which are relative to the animal – it seems logical that the brain would also have neurons sensitive to borders located at particular egocentric directions. Indeed, these have now been reported in several brain regions that lie on the path between primary sensory cortex and the hippocampal place system. These “egocentric boundary cells” were first reported in lateral entorhinal cortex (Wang et al., 2018) and soon afterwards in striatum (Hinman et al., 2019), retrosplenial cortex (Alexander et al., 2020), and post-rhinal cortex (Gofman et al., 2019), suggesting that these neurons form part of a system that translates environmental features from egocentric to allocentric coordinates (see Byrne et al., 2007, for an extensive discussion of this issue).

One consequence of ego- and allocentric boundary coding is that it could potentially enable determination of the global geometry of a bounded space (square, circle, trapezoid, etc.). Whether such geometry is used in spatial cognition has been a much-debated issue, prompted by the famous observations of Cheng and colleagues that rats in a rectangular compartment ignored local features and used geometry to reorient themselves in order to find concealed food (K. Cheng, 1986; Margules & Gallistel, 1988). They did so even though the features were uniquely informative and the geometry ambiguous, which seems to violate principles of associative learning. This observation led to the hypothesis that geometry is a “module” in the spatial system that takes precedence over other forms of spatial cue such as landmarks. Subsequent findings on this issue have been mixed: for example, geometry does not seem to be a strong cue for HD cells (Knight et al., 2011), although it is able to align place cells in a way that correlates with behavioral alignment (Keinath et al., 2017). Cheng and colleagues ultimately moderated the geometric module hypothesis following extensive review of the literature (Cheng et al., 2013). The question is difficult to untangle because geometry usually also influences the visual scene within a bounded space.

Geometry notwithstanding, boundaries are clearly important for cognitive mapping in rodents. Furthermore, recent data from single neuron recording in humans suggests that even “boundaries” in the sequential flow of experience may be encoded by hippocampal neurons (Zheng et al., 2022), perhaps in service of organizing episodic memory (Ross & Easton, 2022). It remains to be determined how the properties of boundaries – namely, their impediment to travel, their extendedness, or the discontinuity they provide – are processed.

### Context

One of the most salient features of place cells is their tendency to alter their firing patterns in response to changes in the environment, called “remapping” as mentioned earlier. This phenomenon was first reported by Muller and colleagues (Muller & Kubie, 1987), who found that transferring the animal to a new environment would cause a reorganization of the firing patterns whereby cells would stop firing, and/or new cells would start firing, or else cells would continue firing but in a different relative location. Because a given neuron participates in many maps in different environments (Alme et al., 2014), this means that the map is a population code, in which each place is represented not by a single cell but by an ensemble of them.

Changing non-spatial characteristics of an environment can alter place fields (Anderson & Jeffery, 2003) even though, as we saw in the preceding section, the cells use the structural components of the environment – the walls – to position their fields. This indicates a dissociation between *whether* a cell fires and *where* it fires. Place cells are thus responding to a combination of spatial and non-spatial cues, which collectively are often called “spatial context” (Bilkey, 2007; Jeffery et al., 2004; Mizumori et al., 1999; Myers & Gluck, 1994; Oler & Markus, 2000; Russell et al., 2003; Sharp, 1999; Smith & Mizumori, 2006). Spatial context is what drives the map selection process described above. The question of what context comprises is at least as complex as the question of what spatial information drives place cells. For example, it has been found that changing the task the animal performs in an environment can alter place fields (Markus et al., 1995), as can changing the sensory modality by which the animal self-localizes (Geva-Sagiv et al., 2016).

Context selects which of a place cell’s potential fields are expressed in a given environment (Hayman & Jeffery, 2008), and as mentioned above, it can be experimentally dissociated from the boundary-related process that determines the precise positioning (Jeffery & Anderson, 2003). This has led to the proposal that contextual inputs “gate” (block or let through) the spatial inputs (Jeffery et al., 2004), perhaps via the grid cells (Hayman & Jeffery, 2008). Context gating can explain both global remapping, in which all cells react together following a context change, and partial remapping, in which only some do, as discussed in more detail later. It can also explain a third type of remapping known as rate remapping, in which cells don’t alter their firing locations but do alter their firing *rates* (Hayman et al., 2003; Leutgeb et al., 2005). Rate remapping could arise if context influences the cells in a graded rather than all-or-nothing fashion, ramping inputs up and down (i.e., acting less like an on-off switch for place fields and more like a dimmer switch).

Contextual cues play more than one role in the assembly of the cognitive map. As well as gating place field expression, context also shapes how head direction cells use environmental features. For example, in one context the cells can use environment layout to indicate North and in another context can use the same layout to indicate South (Jacob et al., 2017). Context can thus influence both positioning *and* orientation of place fields at the same time, via different routes (Cheng et al., 2023).

The route(s) for contextual information to reach the place cell system have not yet been identified, but a prime contender is retrosplenial cortex (Corcoran et al., 2011; Miller et al., 2014; Smith et al., 2012; Trask & Helmstetter, 2022; Vedder et al., 2017), which receives a wide variety of sensory inputs, and which has been shown in behavioral and physiological experiments to be involved in context-processing (reviewed by Mitchell et al., 2018, and Vann et al., 2009). Another candidate is entorhinal cortex, which is the final common pathway for most of the cortical inputs to the place cell system. Given the importance and heterogeneity of contextual information it is likely that there are several routes, cortical and subcortical.

### Compass direction

One of the most important organizing inputs for the cognitive map is the internally constructed compass signal, which we could call the “cognitive compass,” which represents estimated facing direction and is conveyed through the HD system. Heading determination is a primordial signal and was likely among the first spatial competences to arise in evolution, because there are simple sensory cues to global compass direction available in the form of the Earth’s geomagnetic field, and also different sky lighting between North and South. More locally there are directional signals arising from prevailing winds, large-scale olfactory gradients, and so on. Considerable ethological research suggests these most or all of these cues are used for long-range migration, in different ways by different species (Mouritsen, 2018).

The rodent cognitive compass, however, does not use these global, geocentric signals when the animal is indoors: the signal is shaped by immediately local environment cues instead. This can be shown by rotating a cue card in an otherwise symmetrical environment and observing that the cells use the card to break the symmetry and set their orientation each time the animal enters the environment (Taube et al., 1990b). This setting of the HD signal by the cue card only occurs if the environment is familiar, however, and the cells have “learned” the relationship of the cue card to their own intrinsic firing organization. If the environment is unfamiliar then the cells bring with them the orientation they had in the previous environment, sustained by the internal “sense of direction.” However soon thereafter they become controlled by the newly encountered cue card (Taube & Burton, 1995). There is therefore some kind of learning process that anchors environmental cues to a given orientation of the HD cell ensemble.

The cognitive compass (head direction signal) is important for organizing both place and grid cell firing. As we saw earlier, rodent place cells position their fields using the boundaries as guides, and to do this they need to know which wall is which. If there is visual directional information available in the form of a cue card then rotating the cue card causes place cells to rotate their fields just like HD cells do (Muller & Kubie, 1987). If there are two identical cues on opposing walls then the cells can break the symmetry by using the entry point into the environment to initialize the system (Sharp et al., 1990). In situations where the walls are featureless then the only remaining information is the compass direction supplied by the HD cells. This can be shown indirectly by rotating the internal direction sense of the rat and observing that place cells rotate their firing fields accordingly (Jeffery & O’Keefe, 1999).

It was long assumed that the HD compass signal is coherent throughout the network, so that only a single estimate of facing direction is passed to the cognitive map. However, recently a sub-class of HD cells has been discovered that decouples from the “consensus” HD signal and follows the environment layout instead (Jacob et al., 2017; Zhang et al., 2022). These neurons, called “multidirectional” (MD) because they fire in a different global direction in each of several environments, are found in the dysgranular region of retrosplenial cortex, which has strong connections with primary visual brain areas (Van Groen & Wyss, 1992) and has been linked to the processing of landmarks and landmark stability (Auger et al., 2012). The co-existence of HD and MD cells might function to allow the animal to simultaneously both learn about the directional significance of landmarks, and also (once this is learned) use those landmarks in re-orientation (Page & Jeffery, 2018).

As noted earlier, HD cells are also sensitive to context, whereas MD cells are not (because they ignore context to follow the environment layout). This variety of signals in RSC might allow incoming sensory information about environment layout to be mixed with information about context that has been fed back from other regions, most notably hippocampus, in order to reconcile ambiguous inputs and generate an overall heading direction estimate. The output to the cognitive map seems to be a unitary estimate of heading, since the ambiguity expressed by the MD cells in RSC evidently does not affect the place cells (Cheng et al., 2023).

The question arises as to how the cognitive compass operates in three-dimensional (3D) space. A fully 3D compass would not be a simple extension of the functionality that we have discussed for two-dimensional (2D) experiments, because in three dimensions there are two additional planes of rotation. Ordinary rotation to the left or right in the horizontal plane (or the plane of the body – mathematically, it does not matter which reference frame is used) is called yaw. With the addition of the vertical dimension the animal can also rotate in the head-over-heels plane, called pitch, and it can roll to the left or right. These additional possibilities greatly extend the amount of representational capacity needed to process the information. However, because of gravity (discussed in the next section) animals tend to occupy only a small subset of all the possible directions in 3D space and so maintaining capacity to represent all the possibilities is wasteful. This has led to proposals that the cognitive compass in 3D is actually just a modified version of a 2D one, using gravity as an organizing signal, rather than fully 3D. This is explored in the next section.

### Gravity

Gravity is a form of direction signal but it operates only in one dimension, signaling up versus down. It can be detected directly, by the vestibular system, or indirectly for aquatic animals via hydrostatic pressure. Gravity is important for cognitive mapping two main reasons. The first is that it greatly constrains how an animal is able to move and what might happen to it, which is relevant for navigational planning. Movement implications are significant for a heavy animal: it costs effort and energy expenditure for it to move against gravity, and stepping off a cliff can have life-changing consequences. The second is that gravity is spatially symmetry-breaking and is therefore a useful signal for organizing the cognitive map. Imagine an animal floating in space for example, and perceiving only two point landmarks: a red light and a blue light. Given a particular egocentric relationship of the landmarks, the animal could be located anywhere on a ring, in the 3D space. The addition of a gravity vector (or indeed any kind of non-collinear vector) constrains its possible position to a single point.

**Fig. 3:**
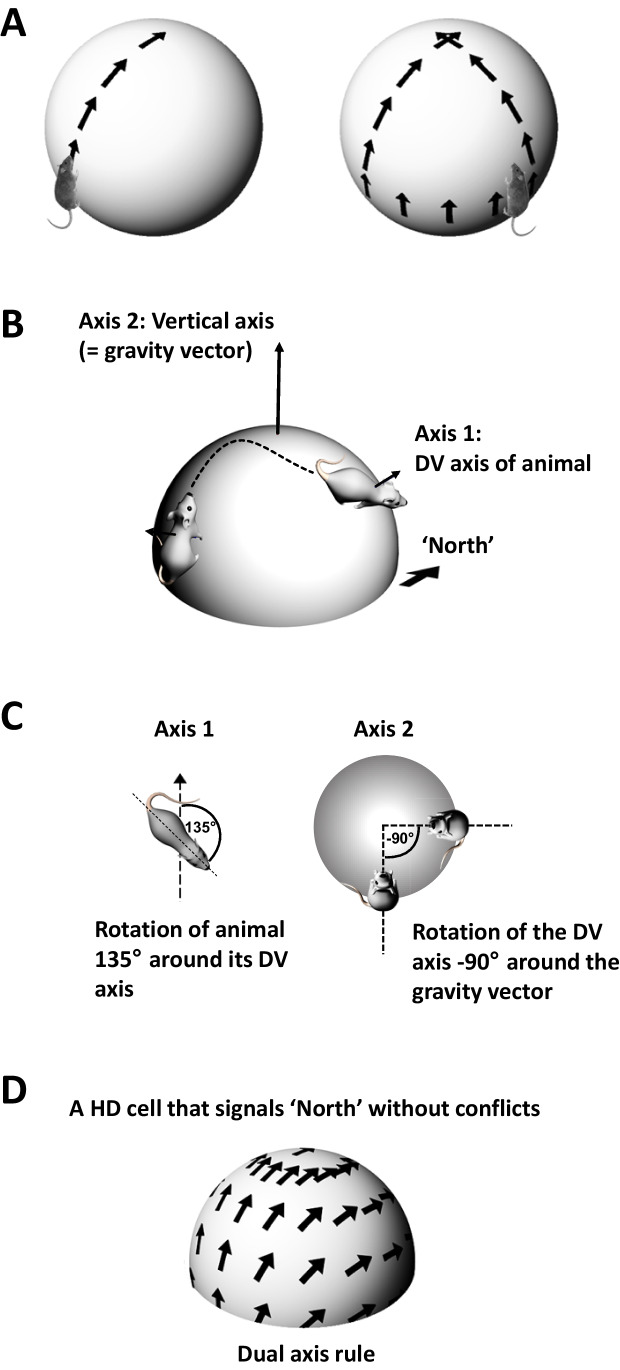
The dual axis rule for updating HD cells in three dimensions. (**A**) The problem: on a three-dimensional surface, a HD cell responsive only to rotations of the head in the plane of the animal could end up firing in a different direction at the same location, depending on the route the animal took to get there. In this example, if an animal walks in a straight line to the top of the ball (left) the HD cell always fires in the same direction because the animal did not turn its head. Similarly, if it first sidles around to the side (right) before ascending then the cell also does not accommodate the change in direction because the animal has also not turned its head in the plane of its body. However, the sidling changed the overall orientation of the plane of the animal relative to the world. The cell thus fires in a different direction when the animal reaches the top of the ball. (**B**) This problem can be avoided if the system can detect the change in direction that occurred during the sidling process, by tracking the change in the pointing direction of the animal’s dorso-ventral (DV) axis (equivalent to its body plane). (**C**) This requires two update rules for HD cells: one tracking rotation around the DV axis in the usual way, and the second tracking rotation of the DV axis around the gravity vector (vertical axis). The dual axis rule simply adds these two rotations. (**D**) When the dual axis rule is applied to the situation in (**A**), now the cell has a smoothly changing firing direction over the surface of the ball, and does not create errors

The constraint imposed by gravity also could be useful for a compass signal. As noted in the preceding section, animals tend to remain horizontal because of how gravity restricts their movement possibilities, and so they do not need complete representational capacity for all possible orientations in 3D space. Furthermore, the gravity signal is a symmetry-breaking cue that could enable a two-dimensional compass to meet all of an animal’s practical needs. How this could work was simultaneously proposed by Page et al. (2018) and Laurens and Angelaki (2018), who suggested that the combined operation of two “flat” compasses, one of these organized around the gravity vector, could together provide an effective 3D compass. In this scheme one of the compasses tracks egocentric yaw (rotation of the animal in its body plane) and the other tracks rotation around the gravity vector of the animal’s dorso-ventral axis (Fig. 3), which is meaningful for every orientation except upright. The two compasses are combined by simply adding the signals together. This scheme ensures that there is a unique state of the system for every upright 3D direction (inverted is more complex to solve) and also, that when the animal returns to upright – say, by reaching the top of a hill – its HD system signals the correct orientation (called “azimuth”) for the horizontal plane. Situations in which this “dual axis” or “tilted azimuth” rule might fail include when the animal is inverted, in which situation rat HD cells, interestingly, lose their directionality (Calton & Taube, 2005). Experimental evidence for the operation of a dual axis compass versus a 3D one is still emerging, and there is evidence for both types of encoding in different species (Finkelstein et al., 2015; Laurens et al., 2013; Shinder & Taube, 2019), although a fully 3D compass in any vertebrate now seems unlikely.

Gravity also seems to have effects on linear spatial processing. Experiments with environments where animals can climb have found that the place and grid cell signals become altered, although the nature of the alteration seems somewhat variable. Grid cells on a steeply tilted surface behaved just as they do on the flat (Hayman et al., 2015), but if the surface was tilted all the way up to vertical, such that the rats had to cling to chicken wire to move around, then the grid fields expanded and the usual regular hexagonal pattern seemed to break down (Casali et al., 2019). In addition, the speed signal that is also expressed in mEC was blunted, suggesting that the cells were receiving reduced information about speed, and hence distance travelled, potentially accounting for the scale expansion. Interestingly, place cell firing fields did *not* expand, but cells were much less likely to have fields on the wall. It seems therefore that it matters for place and grid cells whether a surface is horizontal or vertical. It remains to be determined exactly what it is about the vertical plane that alters the activity.

Overall, then gravity plays an important role in shaping the cognitive map, both in how it affects movement, but also in how it breaks the symmetry of the space and therefore reduces ambiguity.

## Structure of the cognitive map

Having reviewed some of the organizing factors for the cognitive map we turn now to the question of how the map is structured, beginning with a theoretical review of the possibilities. A map, as noted earlier, is a representation that has some kind of correspondence with the thing it is a map of (Gallistel, 1990; Shea, 2014). In the case of real-world space, a cognitive map is an internal “structure” (structured in both space and time) that mirrors the outside world to the extent that some operations on the map reflect operations in the real world. For example, in a spatial map, two adjacent places in the real world should have this adjacency somehow reflected in the map, such that when the map is interrogated (e.g., by some other part of the brain) then the adjacency is revealed and the information can be usefully used. This means that in a familiar environment the animal can know about the adjacency by asking its brain, without having to discover it by walking. The question here is: what aspects of the real world *are* mirrored in the structure of the cognitive map? Is it just adjacencies, or is the map also metric?

A map consisting mainly of adjacency relationships is called topological. A good example of a topological map is the London “Tube map,” which represents locations in London connected by their respective Tube (underground train) lines. The map is not to scale: there is no proportional relationship between distances on the map and distances in the real world. Instead, places are represented relative to each other (next to, between, etc.) with only a loose directional relationship: enough to allow a navigator to move from one broad region to the next, and to know roughly how far to go, but not to precisely localize themself. A metric map, by contrast, is one that *is* to scale: proportional distances are accurately represented (to some defined degree) in both linear and angular domains. A sailor's chart or the map on a mobile phone are good examples. This metric property means that the map can be used with a high degree of precision, for example by using the known distances and angles between landmarks to triangulate one’s current position.

If the map *is* metric, then are these metric properties global, being the same in all environments (like our own artificial maps are), or do properties vary between environments? Are they the same in all dimensions: for example, does the map have the same metricity and same resolution in the vertical dimension as it does in the horizontal (that is, is it isotropic) or do horizontal and vertical differ (anisotropic)?

Next, we can ask whether the map is continuous, or fragmented like a mosaic. If it is continuous then the same type of metric relationships would hold between any two places on the map no matter how far apart they are. If fragmented, then relationships might be consistent within a fragment but differ between fragments. For instance, one could imagine a map structure in which a local, bounded space is metric, but the relationship between the fragments is topological (see Yeap, 1988, and Poucet, 1993, for a discussion of this dual-coding hypothesis). The distinction between metric and topological need not be distinct and indeed is likely more of a continuum. Furthermore, between fragments there might be no spatial relationship at all – the relatedness could be in some other domain entirely, such as action. Many Londoners, for example, have a mosaic-type mental map of London in which they can find their way on foot around a local area but take the bus or Tube to a more distant area: although they can navigate there effectively, they might not even be able to point vaguely in the direction of the second area from the first. Even if the map fragments all have the same metricity, they might be distinguishable by some other property. For example, there is evidence that two adjacent bounded regions, connected by a doorway, can be distinguished by how strongly a given episodic memory is recalled in each space (Radvansky & Copeland, 2006).

A third question about the cognitive map is how it scales across environments of different sizes. Are place fields, for example, the same size in every environment or are they larger in larger spaces? Do they scale with body size?

The final structural question we consider is whether the map is unitary or overlaid. If a map is unitary then each location on the map is represented just once. If overlaid, then each location in physical space has multiple representations, comprising, for example, differently scaled maps, or maps with different properties (such as a terrain map vs. food sources map) or functions (daytime vs. nighttime map). Below, we review the studies from rodent single-neuron investigations that uncovered properties of the mapping of simple space, in conjunction with the later studies that began to explore what happens when spaces become larger and more complex.

### The map is fragmented

**Fig. 4:**
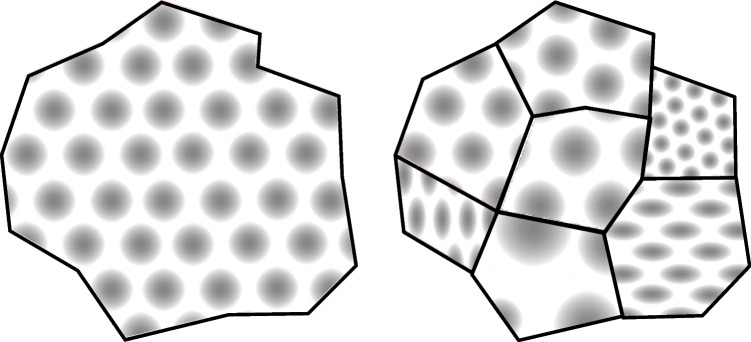
Is the cognitive map unitary or fragmented? **Left:** A unitary map would maintain continuous properties (illustrated here with a hypothetical grid field) right across the space, whereas a fragmented, or mosaic, map (**right**) would be composed of sub-compartments with possibly differing metric properties, and a representation that is discontinuous at the boundaries

The first question we examined above concerning the structure of the cognitive map is whether the map is continuous across connected spaces, or whether it is fragmented (Fig. 4). That is, are there discontinuities, or is it all just One Big Map?

One way to address this question experimentally is to see whether place fields are continuous on either side of environment boundaries. The very early experiment of Muller and Kubie (1987) described earlier found that placing a barrier across the location of a place field attenuated the field and caused the cell to remap. The experiment we also discussed earlier in which walls were progressively removed from a bounded space (Barry et al., 2006), allowing the animal for the first time to explore both sides of the remaining walls, did not see persistence of the fields on the far side of the walls. This was generally true also in a later experiment in which a single wall was removed, allowing the animal to explore the far side of the remaining ones (Rowland et al., 2011). These observations suggest that the map is interrupted at the boundaries of a local space, lending support to the notion that the map is fragmented, with the fragments being defined by local boundaries. Whether this is true for species inhabiting less obviously bounded environments (whales, for example) remains to be determined.

Another way of looking at the continuity of the cognitive map is to take advantage of grid cells. If the map is continuous then arguably so should the grid pattern be, whereas if the map is fragmented, and the fragments are delineated by physical boundaries, then the grid pattern should interrupted at the boundaries. An experiment by Carpenter et al. (2015) seemed to suggest the latter, at least when animals are relatively new to the environment: grid patterns in two adjoining sub-compartments stopped at the boundaries, with the box pattern repeating across the two compartments. This seems slightly surprising, given the presence of the path integration signal that drives the cells, and which should tell the system the chambers are different. In fact, over time the pattern did slowly adapt until it was more continuous across the two spaces. It may be, therefore, that with experience, slow plasticity can convert a fragmented map to a more continuous one. Further work is required to determine whether this continuity aids longer-range navigational planning (presumably so).

Further evidence in favor of the continuity of the cognitive map was provided by observations of head direction cells. Firing directions in different environments can be different, suggesting that the map is fragmented. However, firing directions align if animals can walk freely between the sub-compartments. This was shown by Taube and Burton (1995), who found that the firing direction in a new environment was the same as in the previous one, provided the animal locomoted under its own volition and without disorientation. This alignment is likely supported by a directional path integration, in which angular self-motion cues maintain a stable signal until such time as the cells have learned about the new landmarks. This suggests, as with linear signals, that path integration might at least partly function to “glue” fragments of the map together. Whether initial non-alignment can be slowly corrected with experience, as with the grid cells, remains to be determined.

As well as looking at continuity of cell firing, a way to explore whether the cognitive map is fragmented has been to see whether there are ever “non-local” effects following manipulation to one part of the map – that is, whether changes made in one compartment have an effect on other parts of the map in other compartments. Spiers et al. (2015) recorded place cells as rats explored four identical compartments connected by a corridor. Once the rat was familiarized with the space, they changed one of the compartments from white-walled to black-walled: this induced local remapping, but the effects did not spread beyond the immediate boundaries. They then tried the reverse manipulation of changing every part of the environment *except* that compartment, by removing the internal walls to make a large empty space. This again caused remapping in the changed space, but there was no effect on the unchanged space. Thus, remapping did not spread beyond the local fragment.

If the map is made of multiple fragments, then are the relationships between fragments encoded in the hippocampal place system, and if so, how? That is, does the system “know” that the environment has multiple compartments, or about the order in which compartments are arranged, and whether there is connectivity between them? This is a difficult question to address because it is not clear where and how to look for encoding of places the animal isn’t currently in. One useful tool has been the fact that place cells spontaneously fire in “replay” sequences that re-express the actual sequences of places the animal recently visited (Drieu & Zugaro, 2019). This phenomenon provides an opportunity to investigate connectivity in the spatial map. Using this method, Wu and Foster (2014) found that replay sequences are segmented in the same way as the actual environment is, with sequences stopping at junctions, and varying in length proportionally to the length of the actual maze segments. Replay sequences also flow around barriers (Widloski & Foster, 2022). However, it is still unclear whether this information is meaningful (can be used by the brain to make inferences about the space’s connectivity for planning purposes), or just a reflection of the recent past experience of the animal. By contrast, an experiment in which connectivity was varied between sub-compartments in a multi-roomed environment failed to observe responsiveness of place cells to the changes (Duvelle et al., 2021): the cells did not remap when the doors were open versus closed, despite the large change in relatedness this imposed. So far, there are few data to suggest that the place cells are encoding anything beyond the fairly immediate situation of the animal. However, little work has been done looking at the ventralmost, large-scale place cells in multi-compartment environments: it is possible that the between-compartment continuity of the map (if there is one) will be found here.

### The map is variably metric

If the cognitive map is fragmented, then the next question concerns the metric structure of the fragments: that is, whether/how distances and directions are encoded, and – for multiple dimensions – if this encoding is isotropic or anisotropic.

Studies with place cells suggest that the cognitive map has a metric component and does not arise purely from a confluence of environmental stimuli converging at a particular location. The first evidence came from experiments in which environmental cues were moved, and place fields were found to move incompletely, as though these stimuli were not the only factors driving their positioning. The stretchy box experiment of O’Keefe and Burgess (1996), discussed earlier, found that the movement of place fields was only about 50% of the movement of the walls, indicating some type of countering factor providing a metric input: assumed to be linear path integration. Gothard et al. (1996) moved walls along a linear track while rats were running on it and found that the cells switched between using the external cues and using the internal sense of travel distance (linear path integration again). A subsequent experiment in which self-motion cues were manipulated by having the rat drive a car (as opposed to walking), versus having the environment moved past it, found evidence that three different types of self-motion information – vestibular, locomotor and optic flow – contribute metric information about distance travelled, thus regulating the scale of place fields (Terrazas et al., 2005). These experiments indicate that there is more than one source of distance information being routed into the place cell system. Interestingly, not all place cells respond to all types of information (Chen et al., 2013). Furthermore, for both place and grid cells, experimental alteration of the relationship between visual cues and the physical translation of the animal through space have found that the gain changes, for both place cells (Jayakumar et al., 2019) and grid cells (Campbell et al., 2018), indicating plasticity in the system, which may allow it to be constantly re-calibrated to the environment.

The discovery of grid cells provided important confirmation that the map is metric (Jeffery & Burgess, 2006), because of the regular spacing of the firing fields and the consistent orientation imposed by the path integration process. This convinced many formerly skeptical researchers of the intrinsically spatial nature of the place cell representation. However, very early on, experiments began to suggest that the metric is not rigid. The stretchy box experiment of O’Keefe and Burgess (1996) was repeated with grid cells, with the same result: the grids stretched, but only by about 50% of the box stretching (Barry et al., 2007).

From these findings it was not clear whether this stretchability reflects a normal operating mode of the map (that is, it is at least partly a topological rather than metric map) or whether deformation is a pathological state resulting from an unnatural manipulation. However, subsequent experiments have also found situations in which the grid cell grid does not maintain a stable metric structure even in a non-altered environment. When rats were placed in a novel environment the grid was found to be transiently expanded (Barry et al., 2012). Grid distortion was observed in an irregularly shaped environment even from first exposure (that is, without deforming a familiar one; Krupic et al., 2015). Even in symmetric environments the grid can show distortion (Stensola et al., 2015).

The grid metric sometimes breaks down completely. For example, in situations where the animal is not able to move continuously in a given direction, the grid becomes aperiodic in that direction. This has been seen during running over a two-dimensional surface in the so-called “hairpin maze” (Derdikman et al., 2009), in which rats could only move from one side of the space to the other by running back and forth along corridors aligned in the orthogonal direction (as in an airport security queue). In this apparatus movement was free in one dimension (along the corridors), in which grid cell retained their periodic encoding, but interrupted in the orthogonal dimension, in which they lost it, leading to an overall grid cell pattern that simply repeated the linear component across the space. A similar phenomenon was also seen on a vertical climbing wall in which movement was more difficult and interrupted in the vertical dimension (Hayman et al., 2011; Ulanovsky, 2011). Two recent experiments in rats and bats traversing a volumetric space also found that the metric was disrupted in three dimensions. Traces of it remained: the grid cells still formed constrained firing fields, and although their size was more variable, it was still restricted. Additionally, in the bats, which could traverse the space without interruption (unlike the rats who had to climb on bars), the grid fields had a slightly more consistent spacing than would be expected by chance. It may be that bats and rats have different physiology, but it seems more likely that the difference is due to the different locomotor constraints of the two experiments. In particular, if interrupted travel disturbs grid formation, as suggested by the earlier-described experiments, then the impediments provided by the lattice rungs might have contributed to the greater disorganization of the pattern in rats than was seen in the bats.

The take-home message from this set of experiments is that there is not one single underlying metric structure that can account for all of the patterns seen in these different environments. Rather, the pattern seems to be constructed dynamically, and is shaped by the movement patterns of the animals. We turn now to the issue of larger spaces.

### The map is multiply scaled

A map has a scale, which is the relationship between a unit of coding distance in the map and the corresponding distance in the real world. The scale of the cognitive map is reflected in the size of place fields and the size of the grid cell grids. Oddly, the scales seem consistent across animals of very different sizes: mice and rats do not differ, for example (Fyhn et al., 2008), and nor do baby rats and adult rats (Wills et al., 2010). Experiments with both place and grid cells have revealed that mEC has a range of encoding scales, ranging (in rodents) from small-scale high-resolution encoding in the dorsal region to large-scale lower-resolution encoding in the more ventral regions (Brun et al., 2008; Kjelstrup et al., 2008). Two potential functions of this scale variability have been proposed. One is to enable precise encoding of location. This follows because superimposed grids of different scales yield only a small number of locations where all the fields converge, creating hotpots of activity that may drive place cells (Solstad et al., 2007). The second is to enable computation of distance traveled, using the different scales somewhat like a slide rule (Bush et al., 2015). Whether either of these are the actual function of scale variability (if it has a function) remains undetermined.

What happens if the environment itself enlarges in scale? The answer to this has been relatively little explored relative to the many sizes of environments that are available in nature, largely because most studies are done with rodents, in laboratory settings in which space is necessarily confined. The findings are slightly mixed. The first laboratory manipulation of environment scale was undertaken by Muller and Kubie (1987), who doubled the size of a recording chamber and found that some place cells scaled their fields, albeit – importantly – not proportionally, which is reminiscent of the later O’Keefe and Burgess stretchy box experiment and presumably also reflects the concurrent operation of path integration, which provides an additional distance metric that was not altered by the manipulation. Fenton and colleagues recorded in a small enclosure (68-cm diameter cylinder) and large enclosure (140 × 150-cm square) and found, as the paper title helpfully summarizes, “more place cells and multiple, irregularly arranged, and expanded place fields in the larger space” (Fenton et al., 2008). The importance of the “irregularly arranged” aspect of this observation is that grid cells, the putative drivers of place cells, have population vector patterns that notionally repeat, given a large enough space, and so we might have expected some regularity in the resulting place field distribution. However, this space, although large by laboratory standards, was still relatively small. A much larger open field was used by Harland et al. (2021), who found a similar pattern, with no trace of place field repetition.

Other studies have extended the range of travel by using linear track environments. Kjelstrup et al. (2008) recorded as rats ran back and forth along an 18-m linear track, finding that place field sizes extended up to 10m in length in the more ventral regions. Grid cells on this apparatus also showed a dorso-ventral gradient of scale (Brun et al., 2008), with spacings of up to 3 m expressed by cells in the ventral-most regions. On a track that could be progressively extended up to a maximum of 48 m, place cells were found to show a range of field sizes and field numbers (Rich et al., 2014), together with the interesting observation that cells varied in their propensity to form new fields on the newly added track. That is, rather than all cells firing on the new section with equal probability, some cells were highly likely to add fields in the new space (and thus end up with multiple fields) and some were very unlikely to, remaining silent most of the time. Propensity to fire is a stable property of neurons that is related to their excitability and modulated by the reward value of locations (Lee et al., 2020). A theoretical consequence of this variation in propensity is that in any given size of environment there would be cells with only one or a few fields, these cells providing precise locational information even in the largest spaces. Only one large-scale natural environment has been studied to date, in bats flying large distances to a feeding site (Eliav et al., 2021). Place fields ranged in size from a few centimeters to 32 m: the same cell could express more than one scale.

It seems, then, that the cognitive map shows two types of response to environment scale. One is when a familiar environment rescales, in response to which many place cells partially rescale as if they are trying to find a compromise between what the environment cues are telling them and what the path integration system is telling them. This may be mediated by grid stretching, but alternatively the grid response could be fed back from the place cells (or both). The other phenomenon is that in a large novel environment, more cells become active, some active cells express more place fields, and also larger place fields become expressed. Much remains to be determined, however, about how the place cell map works in very large, unbounded spaces. It seems likely that a migrating bird, for example, is not expressing small-scale place fields throughout its flight, although perhaps extremely large ones are possible. As wireless recording technology becomes more sophisticated it will soon be possible to answer these questions.

### The map is overlaid

The final structure we consider is the extent to which the cognitive map is overlaid. An overlaid map is one in which the same place has multiple representations on multiple different maps, perhaps of different scales, or emphasizing different features, like the street versus satellite views on a phone app.

One line of evidence supporting an overlaid cognitive map is that place cells can remap for the same physical location. This happens, for example, following changes to non-spatial aspects of the environment such as color or odor (Anderson & Jeffery, 2003), as we saw in the section on context. Sometimes the map can change even if nothing changes about the environment at all. In old rats, for example, place cells have been observed to suddenly spontaneously remap, a situation called “multistability” (Barnes et al., 1997). Spontaneous remapping has also been observed in young mice (Sheintuch et al., 2020). Place cell maps can also change due to changes in how the animal interacts with the environment: for example when a rat switches the task it is performing (Markus et al., 1995) or a bat switches from vision to echolocation (Geva-Sagiv et al., 2016). Given their propensity for remapping in the same physical space, in some ways it might be better to think of place cells rather as “situation cells,” where a “situation” is a combination of place plus the expected events or significance associated with the place.

Place cell maps can also be overlaid in more intertwined ways, via partial remapping, in which changes to the environment cause only some cells to remap. Partial remapping was first reported by Skaggs and McNaughton (1998), who found that when rats explored adjacent identical square boxes, a subset of the cells remapped. The experiment of Anderson et al. (2003) showing remapping of some cells to color and some to odor is also an example of partial remapping. Partial remapping can be thought of as revealing the co-existence of sub-maps, which encode different aspects of the environment. For example, the subset of place cells that alter their firing when the odor is changed from vanilla to lemon can be thought of as a sub-map that co-exists with the overlapping subset of cells that remap when the color is changed from back to white. However, the relationship between these sub-maps can be rather complex and enmeshed. The Anderson et al. (2003) experiment found that some cells would remap to color only in the presence of one of the odors, for example, so the color and the odor sub-maps interact. We also saw this kind of remapping in the experiment of Hayman et al. (2003), in which some place cells responded to a change in box location only in one of the colors but not the other. This type of “conditional remapping” (remapping to some cues only in the presence of certain others) is what led to the context gating model of place field generation. There seems to be no limit to the granularity of this interaction: it seems likely that, ultimately, we will find as many sub-maps for a given environment as there are situations that pertain there.

Do sub-maps have a function? One possible function is to allow multiple sets of associations, such as what actions should be performed, to be recruited for the same environment. Supportive evidence for this was provided by an experiment in which rats undertook a hippocampal-dependent behavioral task, running to an unmarked goal location on a large arena, while the environment was changed from black to white (Anderson et al., 2006). This change induced partial remapping, and it also induced a partial change in behavior: rats would still run to the goal location after the change, but they showed thigmotaxis (staying near the walls), indicating that they were anxious about the environment novelty. This suggests that the situation-specific knowledge possessed by an animal could be supported by the overlay of multiple maps for a single physical location.

## The cognitive mosaic

The evidence reviewed above suggests that the mammalian cognitive map is a mosaic of highly variable fragments. At least in rodents, the fragments are defined by the boundaries of each local space. The metric properties of the fragments may differ, with precise metrics (signaled by a regular grid) in some settings and very loose metrics (like the variably sized irregular grids in a volumetric space) in others. How the fragments are related to each other remains to be determined. It seems likely that the head direction signal allows for relating of nearby fragments if the animal can move freely between these. Similarly, the distance metric supplied by the grid cells may allow navigational planning to extend across fragments when the animal has enough experience for the grid to have extended across the space. However, there are surely other relating factors such as actions, relationship to a common distal cue, chained topological relationships (A is next to B, which is next to C), etc.

Many questions remain to be answered. To what extent do the properties uncovered by rodent studies extend across species (providing clues to when they emerged in evolution)? What happens in naturalistic environments where terrain is very uneven and boundaries may not be precise, or may be absent altogether? What even *is* a boundary, for a place or grid cell? Are the mosaic fragments organized into a larger map, and if so, how and where is this map stored in the brain? How is the stored information retrieved and used in navigational planning? How does the map of spatial relations supported by the hippocampal system interact with the map of action relations stored in the striatum? How are these relationships updated in memory during learning or if the environment changes? Answering these questions will require detailed, extended naturalistic studies using wireless recording and 3D inertial tracking, so that animals can be studied expressing natural behaviors during their normal daily lives.

## Data Availability

Not applicable.
